# Safety and clinical outcomes of contemporary liposuction techniques: evidence from a systematic review

**DOI:** 10.3389/fsurg.2026.1892614

**Published:** 2026-09-07

**Authors:** Chen Yang

**Affiliations:** Plastic Surgery, Beijing Shijitan Hospital, Capital Medical University, Beijing, China

**Keywords:** body contouring, liposuction, patient satisfaction, postoperative complications, surgical techniques

## Abstract

Cosmetic liposuction is among the most frequently performed aesthetic procedures worldwide, with multiple technique refinements developed to improve efficiency, recovery, and safety. These include suction-assisted (SAL), power-assisted (PAL), ultrasound-assisted (UAL), laser-assisted (LAL), water-assisted (WAL), and radiofrequency-assisted liposuction (RFAL). Comparative evidence focused exclusively on isolated cosmetic cases remains limited. A systematic search of PubMed, EMBASE, and Web of Science was conducted to identify clinical studies on isolated cosmetic liposuction published between 2000 and 2025. Randomized trials, cohort studies, and extensive case series were included. The primary outcome was the incidence of procedure-related complications. Secondary outcomes, including efficacy, patient satisfaction, recovery, skin tightening, and durability, were synthesized qualitatively because of heterogeneity in outcome reporting. Thirteen studies met the inclusion criteria. Only one included study provided fully extractable numerical safety data for overall complications, seroma, and infection. Therefore, these safety outcomes are presented descriptively rather than as pooled meta-analytic estimates. The available evidence indicated low reported rates of overall complications, seroma, and infection, while serious complications remained rare (<0.1%). Qualitative synthesis showed that all modalities achieved comparable fat reduction and contour improvement. Studies generally reported improved operative efficiency with PAL, reduced early postoperative pain and blood loss with WAL, and modest short-term skin tightening with LAL/RFAL. Patient satisfaction was consistently high (≥80%) across techniques. Contemporary cosmetic liposuction techniques demonstrate favorable safety profiles, while evidence regarding efficacy, recovery, patient satisfaction, skin tightening, and durability should be interpreted cautiously because it was derived primarily from heterogeneous qualitative evidence.

## 1 Introduction

Cosmetic liposuction—the elective removal of unwanted fat deposits—is one of the world's most common plastic surgery procedures. Differences in safety, recovery, and durability across techniques may have important implications for patient counseling, operative planning, and risk mitigation (1). Surgeons have responded by creating variations of the original tumescent (suction-assisted) mode, adding adjuncts of ultrasound-assisted (UAL, e.g., VASER®), laser-assisted (LAL), power-assisted (PAL), and water-assisted (WAL) liposuction. All modalities involve mechanical or energy-based forces for the removal of fat and are performed in accordance with the current safety guidelines (e.g., tumescent fluid infusion).

Many studies indicate that liposuction is decreasing treated fat volumes and enhancing body contours. PAL was found to hasten the fat aspiration rate and reduce the operating time (2, 3). Ultrasound-assisted liposuction (UAL, e.g., VASER) fluidizes fat in fibrous areas, easing extraction (4). Third-generation ultrasound-assisted liposuction (VASER) has further improved selective adipose emulsification while minimizing injury to adjacent neurovascular structures. Recent clinical experience indicates that modern UAL systems facilitate treatment of fibrous anatomical regions, improve operative precision, and may reduce intraoperative blood loss compared with conventional suction-assisted liposuction, particularly during large-volume body contouring procedures (5). LAL is often promoted for refinement and dermal heating (skin tightening) (6, 7). WAL uses a pressurized water jet to gently dislodge fat, resulting in less postoperative pain and bruising (8). Despite these differences, most studies report similar total fat removal and high patient satisfaction (≥80%) across techniques (9). All methods achieve significant circumference reduction, and technique selection often reflects the surgeon's experience and patient-specific factors rather than efficacy.

Complication rates are low with modern liposuction when performed by experienced surgeons, although safety profiles differ by technique. A systematic review and meta-analysis comparing isolated aesthetic liposuction techniques reported that LAL had the lowest pooled rates of hemorrhage, infection, seroma, burns/blistering (thermal injury), and cutaneous necrosis (each ∼0.13%). PAL showed higher pooled rates for hemorrhage (0.72%) and infection (1.34%), and the highest pooled cutaneous necrosis (0.72%). Ultrasound-assisted liposuction (UAL) had the lowest pooled rate of contour irregularities (∼0.12%), whereas traditional suction-assisted liposuction (SAL) had the highest pooled contour irregularities (∼3.36%). Radiofrequency-assisted liposuction (RFAL) showed comparatively higher pooled rates of seroma (∼3.93%) and thermal injuries/blistering (∼1.64%). Technique-specific risk awareness is important for patient counseling and operative planning (10).

Across broader liposuction literature, a large systematic review/meta-analysis reported an overall complication rate around 2.62%, with contour deformity being the most common reported event; serious events were rare, including venous thromboembolism and local anesthetic toxicity (11). Available longer-term durability of contour outcomes appears most dependent on weight stability and behavior rather than the device itself. In a randomized trial, abdominal liposuction did not induce regrowth of subcutaneous fat, although compensatory increases in visceral fat were observed (12). In longer-term observational follow-up, reductions in total body fat mass have been reported to persist up to ∼1.5–4 years after large-volume liposuction, though metabolic risk markers may not necessarily improve (13). Patient-reported outcomes also tend to be favorable: a survey-based clinical experience found that ∼80% of patients reported satisfaction and ∼80% would undergo liposuction again, with weight gain cited among factors linked to dissatisfaction or perceived “fat return” (9). Procedure-specific satisfaction has also been reported for energy-assisted approaches such as UAL, where nearly ∼80% of patients described being completely/mostly satisfied (14).

To date, most published syntheses have focused on complication rates, single-device categories, or mixed populations that include reconstructive indications and combined procedures. These approaches limit technique-specific interpretation of efficacy, recovery, and longer-term durability in purely cosmetic settings. Accordingly, a gap remains for a comprehensive synthesis that simultaneously evaluates efficacy, safety, recovery profiles, and longer-term outcomes across all major liposuction modalities within isolated aesthetic populations. Unlike previous systematic reviews, the present review was restricted exclusively to isolated cosmetic liposuction procedures and excluded reconstructive indications and combined surgical procedures to minimize clinical heterogeneity. Furthermore, while safety outcomes were summarized using descriptive safety reporting when extractable numerical data were available, efficacy, patient recovery, skin tightening, patient satisfaction, and longer-term durability were systematically evaluated using a structured qualitative synthesis because of substantial heterogeneity in outcome definitions and reporting across studies. By integrating these outcomes across all major contemporary liposuction techniques, this review offers clinicians with a practical evidence-based framework for selecting the most appropriate modality according to patient characteristics, anatomical site, aesthetic goals, and recovery priorities rather than presumed device superiority. By comparatively evaluating all major contemporary liposuction modalities (SAL, PAL, UAL/VASER, LAL, WAL, and RFAL) within isolated cosmetic populations, the present review extends previous evidence syntheses that focused primarily on complication rates or individual technologies and supports more individualized clinical decision-making in aesthetic liposuction.

## 2 Method

### 2.1 Literature search strategy

A systematic literature review of PubMed, EMBASE, and Web of Science databases from January 2000 through March 2025, using search terms related to liposuction techniques (e.g., tumescent, ultrasound-assisted, laser-assisted, power-assisted, water-assisted, radiofrequency-assisted) and clinical outcomes (e.g., fat volume removed, complications, patient satisfaction, skin tightening) was conducted. The final literature search was conducted on 15 March 2025. The complete search strategies for all databases, including Boolean operators and search terms, are provided in Supplementary Table S1. Reference lists of included studies were manually screened to identify additional eligible articles. The review was registered in PROSPERO (CRD420261304785).

### 2.2 Eligibility criteria

Eligible studies included peer-reviewed clinical investigations in cosmetic populations, including randomized controlled trials, prospective or retrospective cohort studies, large case series, and national surveys reporting safety, efficacy, or patient-reported outcomes following isolated liposuction. Both comparative and non-comparative clinical studies were considered eligible, provided they reported extractable outcome data.

Excluded studies involve disease-specific or reconstructive indications (e.g., lipedema, gynecomastia), combined surgical procedures (e.g., liposuction with abdominoplasty), experimental or *in-vitro* studies, isolated case reports, and non-peer-reviewed sources. Only studies of isolated liposuction in otherwise healthy adult patients were included.

### 2.3 Outcomes

The primary outcome was procedure-related complications. Where extractable numerical event data were available, these outcomes were summarized descriptively. Secondary outcomes, including efficacy, recovery, patient satisfaction, skin tightening, contour quality, and long-term durability, were evaluated through qualitative narrative synthesis because of substantial heterogeneity in outcome definitions, measurement methods, and reporting across the included studies. This structured qualitative synthesis was prespecified to capture clinically relevant outcomes that could not be appropriately combined in a quantitative meta-analysis.

### 2.3 Data extraction

Data extracted included aspirated fat volume, objective contour or skin tightening measurements, patient-reported satisfaction outcomes (including validated and non-validated questionnaires, Likert-type rating scales, structured postoperative surveys, or patient interviews, where reported) or quality-of-life outcomes, and the incidence of procedure-related complications and adverse events (including hematoma, seroma, infection, thermal injury, contour irregularity, skin necrosis, and, where reported, serious complications such as organ injury, thromboembolism, or local anesthetic systemic toxicity). Data extraction was performed by the author using a standardized data extraction form and predefined extraction criteria. In addition, information regarding the surgical approach (superficial, deep, or combined liposuction where reported), device platform, and energy delivery parameters (e.g., VASER probe configuration, ultrasound settings, laser wavelength, radiofrequency system, or water-jet platform) was extracted whenever available. Owing to substantial variability in reporting across studies, these technical details were synthesized narratively rather than quantitatively.

### 2.4 Study selection process

The author independently screened titles and abstracts, followed by full-text assessment for eligibility. Study selection was performed using predefined eligibility criteria in accordance with the review protocol and the PRISMA 2020 guidelines. The study selection process was documented using a PRISMA flow diagram.

### 2.5 Risk of bias assessment

Study quality and risk of bias were assessed by the author using validated risk-of-bias assessment tools appropriate to the study design. Randomized controlled trials were evaluated using the Cochrane Risk of Bias 2 (RoB 2) tool, while non-randomized studies were assessed using the Risk Of Bias In Non-randomized Studies of Interventions (ROBINS-I) tool. Each study was evaluated across the relevant methodological domains specified by the respective assessment tool, and overall risk-of-bias judgments were assigned in accordance with published guidance. RoB 2 judgments were categorized as “low risk,” “some concerns,” or “high risk,” whereas ROBINS-I judgments were categorized as “low,” “moderate,” “serious,” “critical,” or “no information.” The two tools were interpreted separately and were not converted to, or combined using, a common numerical scale.

### 2.6 Data synthesis and statistical analysis

Where multiple studies reported comparable outcomes, results were synthesized qualitatively and, when sufficient study-level numerical event data were available, quantitative synthesis was planned. However, verification of the extracted data demonstrated that only one study provided fully extractable numerical safety data for overall complications, seroma, and infection. Consequently, these outcomes were summarized descriptively rather than quantitatively, and no pooled meta-analysis, forest plots, or heterogeneity statistics were generated. Where quantitative synthesis was not appropriate, outcome ranges or representative values were reported descriptively instead. No prespecified subgroup, sensitivity, or *post hoc* analyses were performed because the included studies provided insufficient stratified outcome data across liposuction techniques to permit statistically meaningful comparisons, and study-level reporting was too inconsistent to support robust secondary analyses. Formal assessment of publication bias was not performed due to the limited number of eligible studies. For each reported safety outcome (overall complications, seroma, and infection), the contributing studies, study design, unit of analysis (patients or procedures), available denominator, and reported adverse events were recorded whenever extractable. Because the available studies reported outcomes using different units of analysis, these data were not combined quantitatively. Instead, each study was interpreted according to its original unit of analysis. Patient-level denominators were preferentially interpreted where available; however, when studies reported outcomes per procedure rather than per patient, the original procedure-level denominator was retained and explicitly identified throughout the manuscript. Split-body studies were interpreted using paired anatomical site-level observations as reported in the original publications and were not combined with patient-level or procedure-level data. Where studies involved multiple procedures or reported more than one complication in the same patient, outcomes were extracted exactly as presented in the original publication without recalculation or conversion between units of analysis.

The certainty of evidence for the primary outcome was assessed qualitatively using GRADE domains, but formal evidence grading was limited by heterogeneity and study design variability. All conclusions were derived exclusively from peer-reviewed clinical publications.

## 3 Results

### 3.1 Study selection and characteristics

**Figure 1:**
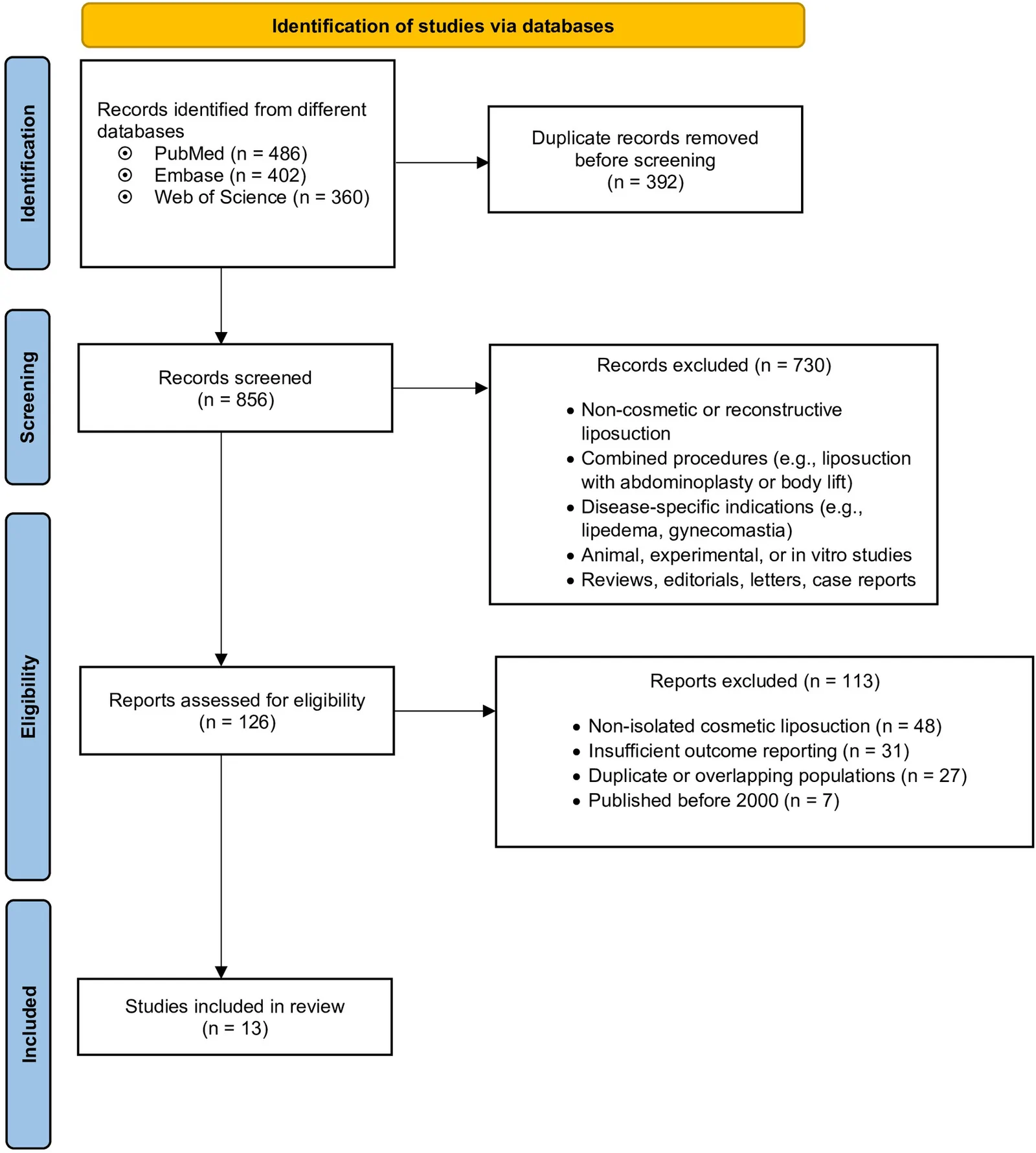
PRISMA flow diagram.

The systematic search of PubMed, EMBASE, and Web of Science identified 1,248 records. After removal of duplicates and title/abstract screening, 126 full-text articles were assessed for eligibility. Studies were excluded if they involved reconstructive or disease-specific indications, combined procedures, experimental models, or insufficient outcome reporting. Seven studies were excluded because they were published before the year 2000 and fell outside the predefined eligibility period. Ultimately, 13 studies met all inclusion criteria and were included in the qualitative synthesis (2, 5, 6, 8, 15–23), with one study providing extractable numerical safety data for descriptive safety reporting (Figure 1).

The characteristics of the included studies are summarized in Table 1. The technical characteristics of the liposuction systems and operative protocols used across the included studies are summarized in Table 2. Considerable heterogeneity was observed in the surgical approaches and device-specific configurations. Most conventional suction-assisted liposuction (SAL) procedures employed the tumescent technique with deep or combined superficial–deep fat aspiration. Ultrasound-assisted liposuction studies primarily evaluated VASER systems; however, probe configuration, ultrasound power settings, pulse mode, and treatment duration were inconsistently reported. Likewise, laser-assisted liposuction studies used different laser platforms and wavelengths, while radiofrequency-assisted liposuction (RFAL) and water-assisted liposuction (WAL) investigations employed manufacturer-specific systems with varying operative protocols. Because these technical parameters were not uniformly described, direct comparison of individual device settings was not feasible.

**Table 1:** Characteristics of included studies.

Author (Year)	Design	Technique	N (patients/procedures)	Primary outcomes	Follow-up
Housman et al. (2002) (15)	National survey	SAL	66,570 procedures	Safety, SAE rate, mortality, complication rates by setting and sedation	Retrospective review of procedures performed from 1994 to 2000 (7-year study period); no patient-specific follow-up reported
Katz et al. (2003) (16)	Retrospective comparative cohort study	PAL	207 patients	Complication rate (systemic and local), safety profile (seroma), comparison with traditional liposuction	Short-term
Scuderi et al. (2005) (2)	Comparative study	PAL vs SAL	15 patients	Efficiency/output (aspiration performance)	Intraoperative (no postoperative follow-up reported)
Prado et al. (2006) (17)	RCT (double-blind)	LAL vs SAL	25 patients	Clinical outcomes + biochemical safety signals	3–5 days, 12–15 days, and 6–11 months
Araco et al. (2007) (8)	RCT	Power WAL vs SAL	60 patients	Pain, ecchymosis/tissue trauma	Early post-op
Roustaei et al. (2009) (18)	Prospective cohort	UAL	660 procedures	Complications, safety	12 weeks
DiBernardo et al. (2010) (6)	Randomized, blinded split-abdomen	LAL vs SAL	10 patients	Skin tightening	3 months (reported)
Sasaki (2011) (19)	Prospective consecutive clinical series	WAL	41 patients	Safety, efficacy, postoperative recovery, and fat-harvesting outcomes	4 weeks
Nagy et al. (2012) (20)	Multicenter RCT (single-blind)	UAL (VASER) vs SAL	20 patients	Skin retraction, blood loss	6 months
Hurwitz and Smith (2012) (21)	Prospective cohort	RFAL	17 patients	Circumference reduction, tightening	3–26 months
Theodorou et al. (2012) (22)	Prospective cohort	RFAL	97 patients	Safety, satisfaction, tightening	6 months
Duncan (2012) (23)	Prospective pilot	RFAL	12 patients	Skin tightening	12 months
Tran et al. (2022) (5)	Retrospective cohort	UAL	261 patients	Safety, efficacy	Short-term

**Table 2:** Technical characteristics of liposuction systems and operative protocols reported in the included studies.

Study	Surgical approach	Device/System	Technical parameters reported
Housman et al. (2002) (15)	Suction-assisted liposuction (SAL)	Conventional suction-assisted liposuction	Device manufacturer, cannula specifications, suction pressure, and operative parameters were incompletely reported.
Katz et al. (2003) (16)	Power-assisted liposuction (PAL) using tumescent anesthesia (compared with traditional liposuction)	Power-assisted liposuction system with a reciprocating cannula	Procedures were performed under tumescent local anesthesia using a reciprocating cannula. Device manufacturer, cannula reciprocation rate, cannula diameter, aspiration pressure, and other technical settings were incompletely reported.
Scuderi et al. (2005) (2)	Suction-assisted liposuction (SAL)	Conventional suction-assisted liposuction	Technical specifications of the device and operative settings were incompletely reported.
Prado et al. (2006) (17)	SAL vs. Laser-assisted liposuction (LAL)	1,064-nm Nd:YAG laser	Laser wavelength (1,064 nm) was reported. Laser-assisted lipolysis protocol was described, although detailed energy delivery parameters were incompletely reported.
Araco et al. (2007) (8)	Water-assisted liposuction (WAL) vs. suction-assisted liposuction (SAL)	Body-Jet® water-assisted liposuction system	Water-assisted liposuction was performed using the Body-Jet® system. Water-jet pressure settings, infiltration volume, aspiration pressure, and other device-specific operative parameters were incompletely reported.
Roustaei et al. (2009) (18)	Ultrasound-assisted liposuction (UAL)	Ultrasound-assisted liposuction (UAL) system	Ultrasound-assisted liposuction was performed under local tumescent anesthesia. The study evaluated the safety of UAL in 660 procedures. Specific device manufacturer, probe configuration, ultrasound power settings, pulse mode, treatment duration, and other technical parameters were incompletely reported.
DiBernardo et al. (2010) (6)	Laser-assisted liposuction (split-body trial)	1,064-nm Nd:YAG laser	Laser wavelength and treatment protocol were reported; detailed fluence and energy delivery settings were incompletely described.
Sasaki (2011) (19)	Water-assisted liposuction (WAL)	Body-Jet®	Water-assisted liposuction was performed using the Body-Jet system with simultaneous infiltration and aspiration. Water-jet pressure settings and other device-specific parameters were not reported.
Nagy et al. (2012) (20)	Ultrasound-assisted liposuction (UAL)	VASER	VASER-assisted liposuction was performed; probe configuration, ultrasound power settings, pulse mode, and treatment duration were incompletely reported.
Hurwitz and Smith (2012) (21)	Radiofrequency-assisted liposuction (RFAL)	BodyTite®	Bipolar radiofrequency-assisted liposuction was performed using the BodyTite system. Radiofrequency energy was applied according to the study protocol; detailed power settings and treatment parameters were incompletely reported.
Theodorou et al. (2012) (22)	Radiofrequency-assisted liposuction (RFAL)	BodyTite®	BodyTite radiofrequency-assisted liposuction was used. Detailed radiofrequency energy settings, treatment duration, and device parameters were incompletely reported.
Tran et al. (2022) (5)	VASER-assisted liposuction	Third-generation VASER®	Third-generation VASER ultrasound-assisted liposuction was performed using manufacturer-recommended operative protocol for the third-generation VASER system. Specific probe configuration, ultrasound energy settings, pulse mode, and treatment duration were incompletely reported.

The evidence base comprised randomized controlled trials, prospective and retrospective cohort studies, comparative clinical studies, pilot investigations, and national surveys. Following verification of the original publications, Sasaki (19) was reclassified from a randomized controlled trial to a prospective consecutive clinical series. Sample sizes ranged from 10 patients (with paired anatomical site-level assessments in split-body randomized studies) to 66,570 procedures in a national survey. Accordingly, the unit of analysis varied across studies (patients, procedures, or anatomical sites), depending on the original study design and reporting. Follow-up durations also varied considerably, ranging from intraoperative assessments and early postoperative evaluations to 12 months, with one national survey reporting procedural outcomes collected over a seven-year period. The evaluated techniques included suction-assisted liposuction (SAL), power-assisted liposuction (PAL), ultrasound-assisted liposuction (UAL), laser-assisted liposuction (LAL), water-assisted liposuction (WAL), and radiofrequency-assisted liposuction (RFAL).

**Figure 2:**
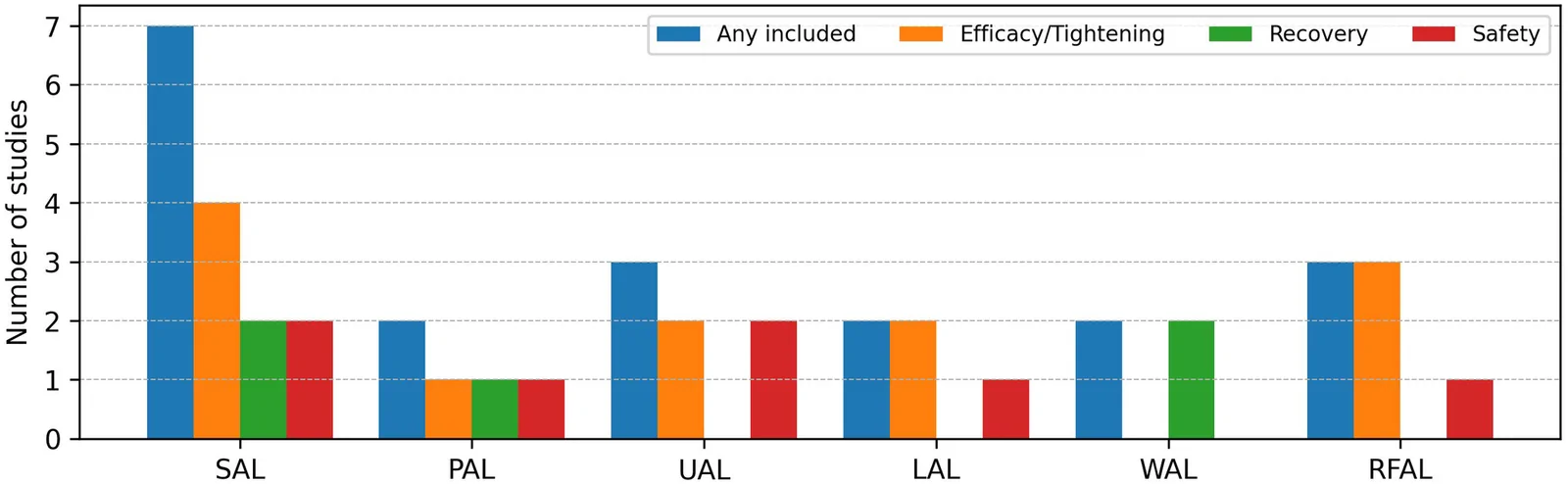
Distribution of included evidence by technique and outcome domain.

Figure 2 shows the distribution of the included studies by liposuction methods and outcome domains (efficacy/tightening, recovery, and safety). SAL evidence was most numerous, especially on efficacy and safety outcomes, but recovery-oriented outcomes were more commonly published in WAL research. Energy-assisted methods were found to have a more balanced but smaller evidence base, in favor of narrative synthesis of selected outcomes.

**Figure 3:**
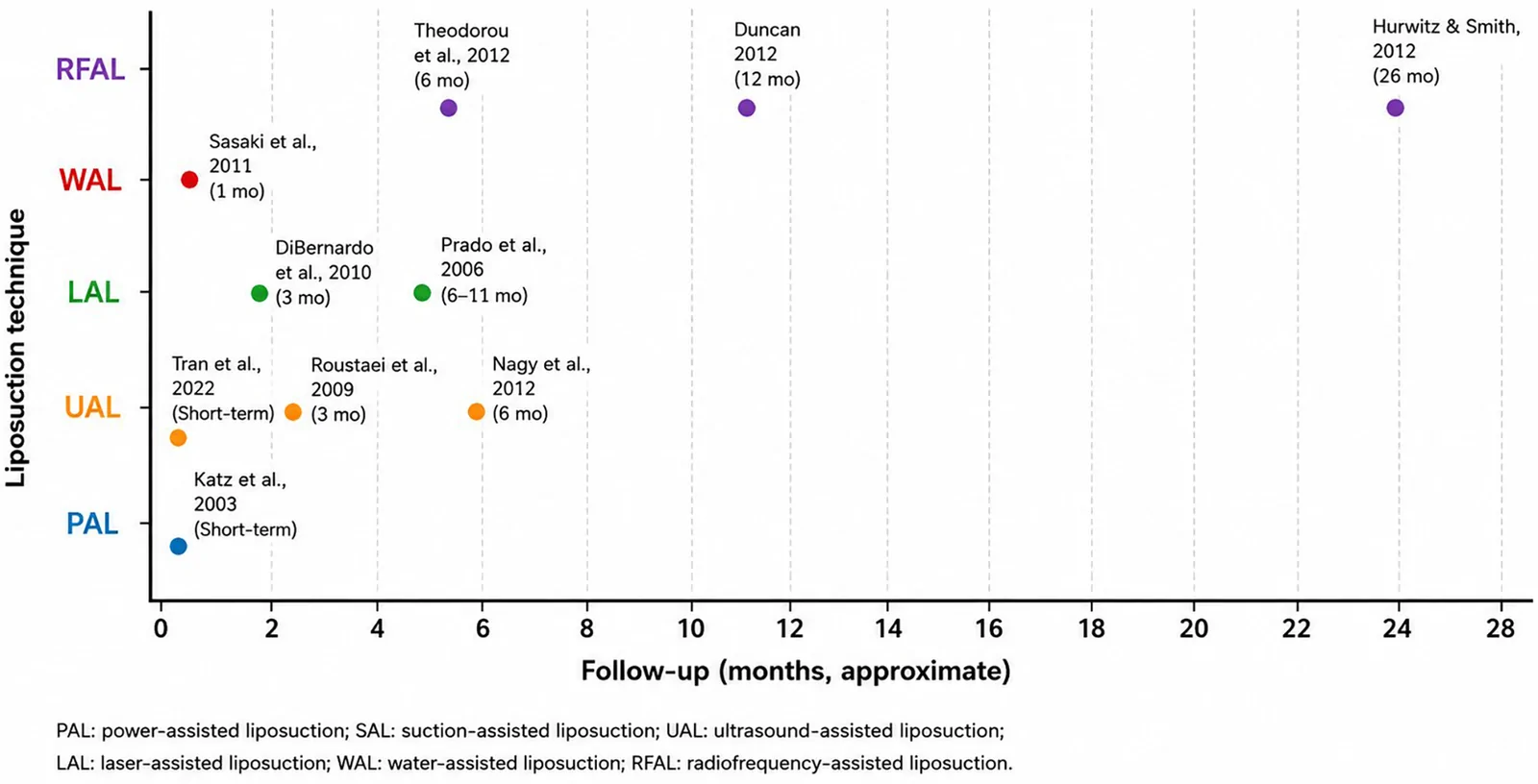
Reported postoperative follow-up duration by liposuction technique among the included studies.

The duration of reported follow-up varied substantially across studies, ranging from intraoperative assessments and 3–5-day postoperative evaluations to 12-month follow-up, while the national survey summarized procedures performed over a seven-year study period without patient-level longitudinal follow-up. Figure 3 illustrates the reported postoperative follow-up duration among studies providing follow-up data, demonstrating generally shorter follow-up periods for WAL, PAL, and UAL studies and relatively longer follow-up among RFAL studies.

The distribution of evidence across techniques and outcome domains (efficacy/tightening, recovery, and safety) is summarized in Table 3.

**Table 3:** Distribution of evidence by technique and outcome domain.

Technique	Outcome domain	Number of studies
SAL	Efficacy/Tightening	4
SAL	Recovery	2
SAL	Safety	2
PAL	Efficacy/Tightening	1
PAL	Recovery	1
PAL	Safety	1
UAL	Efficacy/Tightening	2
UAL	Recovery	0
UAL	Safety	2
LAL	Efficacy/Tightening	2
LAL	Recovery	0
LAL	Safety	1
WAL	Efficacy/Tightening	0
WAL	Recovery	2
WAL	Safety	0
RFAL	Efficacy/Tightening	2
RFAL	Recovery	0
RFAL	Safety	1

### 3.2 Efficacy and contouring outcomes

**Figure 4:**
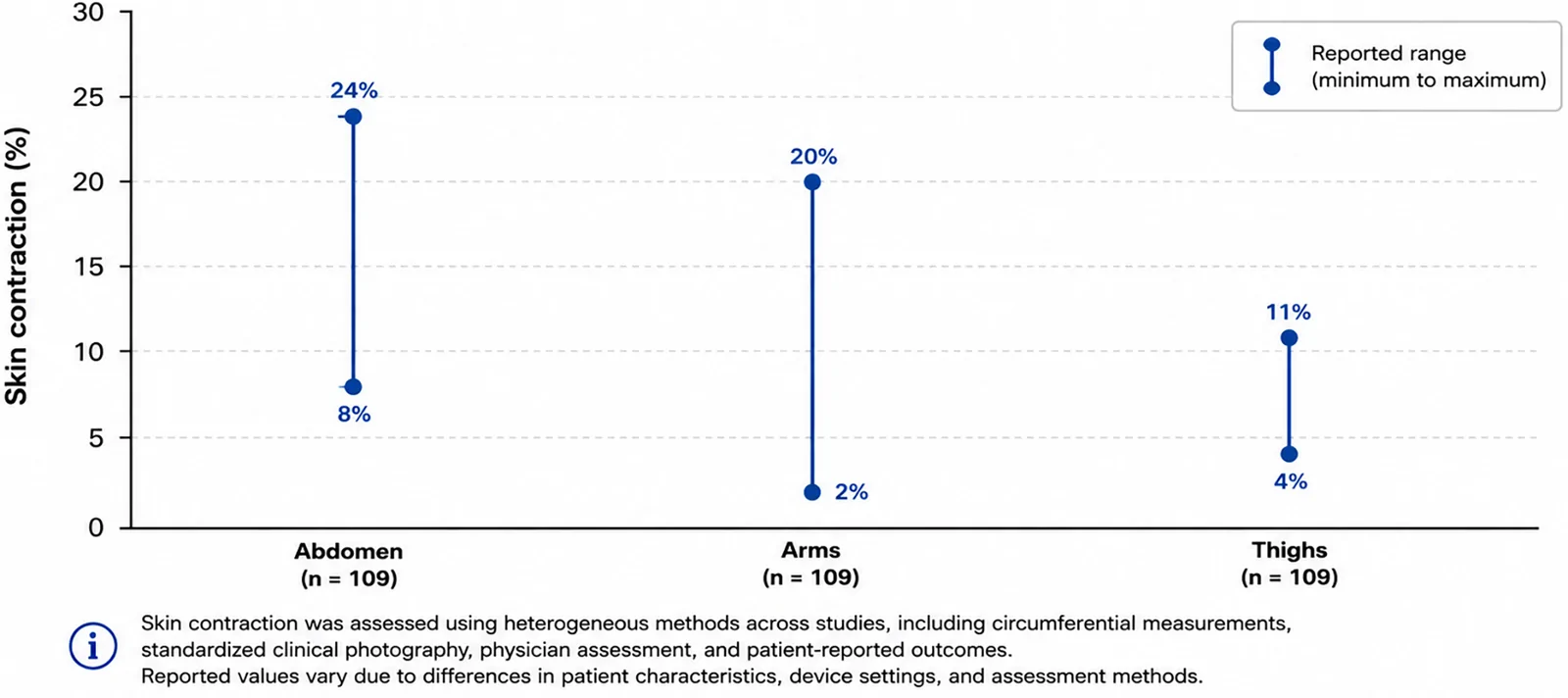
Summary of reported skin tightening following RFAL by anatomical site.

In all studies incorporated in the review, no method of liposuction showed consistent effectiveness in weight loss in terms of total fat loss in cases where the procedures were carried out within the safety boundaries. Comparative analyses have consistently demonstrated that the efficacy was motivated by surgical indication and technique performance as opposed to the type of device. PAL studies showed much better aspiration efficacy, and greater fat aspirated per unit time, and lesser time of operation, without any significant variations in final contour outcomes. Fibrous or secondary areas of treatment had reported smoother passage of the cannula in UAL and LAL studies, and lower tissue resistance, but postoperative circumference reduction and contour were similar to SAL. RFAL trials revealed consistent circumference reduction and clinical skin tightening throughout follow-up (3–26 months). Skin tightening outcomes were assessed using heterogeneous methodologies across studies, including standardized clinical photography, circumferential body measurements, investigator-based aesthetic assessment, patient satisfaction questionnaires, and, in selected studies, quantitative measurements of skin tightening. Because objective assessment methods were not standardized, direct comparison of skin-tightening efficacy between technologies should be interpreted cautiously. The reported mean skin tightening affected the reaction, depending on the anatomical location, with the abdomen recording the highest reaction. Figure 4 gives a summary of the reported RFAL skin tightening results by anatomical site. Table 4 presents a summary of clinically oriented, consolidated comparisons of liposuction methods, such as the best use and application of the method, the benefits, and the constraints, as well as general confidence in the evidence reliability of the method.

**Table 4:** Comparative summary of liposuction techniques.

Technique	Best clinical use-case	Main findings from the included studies	Key limitations/risks	Evidence base in this review	Overall certainty (qualitative)
SAL (tumescent)	Baseline comparator; broadly applicable	Consistently demonstrated acceptable safety and effectiveness across diverse cosmetic indications; frequently served as the reference technique.	Operator fatigue; contour irregularity depends on the technique	Survey/cohorts; comparator arms	Moderate
PAL	Efficiency-focused suction	Studies generally reported faster aspiration and reduced surgeon fatigue, particularly in larger-volume procedures.	Device cost; minor complication reporting varies	Case series + comparative performance	Low–Moderate
UAL (VASER)	Fibrous areas/secondary lipo; potential reduced blood loss	Studies suggested improved tissue handling in fibrous regions and potential reduction in blood loss.	Thermal injury risk if misused; learning curve	Prospective cohorts + multicenter RCT + large series	Moderate
LAL	Small areas; mild-to-moderate laxity (adjunct tightening)	Several studies reported short-term skin tightening and coagulative effects; evidence for superior overall efficacy remains limited.	Burn risk; benefit mainly tightening rather than extra fat removal	RCTs including split-area designs	Moderate
WAL (Body-Jet)	Recovery-prioritized; gentle harvest	Randomized studies generally reported less early postoperative pain and bruising with faster early recovery.	Specialized equipment; long-term contour data limited	RCTs focused on recovery outcomes	Moderate
RFAL	Patients with laxity where tightening is desired	Prospective cohort studies reported consistent short-term skin tightening and circumference reduction; higher seroma reporting has been observed in some cohorts.	Energy-related events (seroma/burn) reported in broader literature; operator-dependent	Prospective cohorts + pilot study	Low–Moderate

### 3.3 Patient recovery and satisfaction

The highest frequencies of recovery outcomes were reported in WAL and PAL studies. Comparative randomized trials in the use of WAL vs. SAL all showed a statistically significant reduction in pain, bruising, and ecchymosis, and quicker recovery to normal activity, in the initial weeks of the postoperative period. PAL was linked to small decreases in postoperative pain, probably the result of reduced operative duration. In all methods, patient satisfaction was generally consistent at around 80% or higher, although satisfaction was assessed using heterogeneous instruments across studies, including patient satisfaction questionnaires, Likert-type rating scales, structured postoperative surveys, and patient interviews. Because these assessment methods were not standardized, direct quantitative comparison between techniques was not feasible. There were no long-term variations between modalities after the healing had taken place. The correlation between satisfaction and smooth contour outcome and proper patient selection seemed higher than that between satisfaction and device choice.

### 3.4 Safety outcomes

Safety outcomes were reviewed to determine their suitability for quantitative synthesis. Following verification of the extracted data, only Roustaei et al. (18) provided fully extractable numerical event counts for overall complications, seroma, and infection. Therefore, these outcomes are reported descriptively rather than as pooled meta-analytic estimates. Supplementary Table S2 summarizes the studies reporting safety outcomes, identifies those with extractable numerical data, specifies the original unit of analysis, and clarifies that descriptive findings were interpreted according to the original reporting without combining different analytical units.

In the single study providing fully extractable numerical safety data, seroma and infection were reported infrequently. Serious complications, including organ injury, thromboembolism, and local anaesthetic systemic toxicity, were reported only rarely across the included studies. However, because these events were inconsistently reported and study-level event counts and denominators were not uniformly available, their frequency could not be estimated reliably or compared between techniques. Because only one study provided fully extractable numerical safety data for these endpoints, pooled estimates, forest plots, and between-study heterogeneity statistics were not generated. Given the limited number of studies reporting extractable safety outcomes and the substantial variability in study design and outcome reporting, no subgroup or sensitivity analyses were performed. Descriptive trends across the included studies suggested that LAL was generally associated with lower reported complication rates, whereas PAL and RFAL were associated with slightly higher reporting of minor complications and seroma, respectively. However, these observations were derived from a limited number of heterogeneous studies and should not be interpreted as definitive comparative differences between techniques.

### 3.5 Certainty of evidence (GRADE assessment)

The GRADE framework was used to evaluate the certainty of evidence for the primary outcome (overall complication rate). The certainty of evidence for quantitative safety outcomes was judged to be low because only one study provided fully extractable numerical event data, precluding assessment of consistency and statistical heterogeneity across studies. The certainty of evidence for secondary outcomes, including recovery, efficacy and longer-term durability, was low to very low because of indirectness, variability in outcome definitions and measurement methods, limited follow-up duration, imprecision resulting from small event numbers, and inconsistency in reporting, including differences in the unit of analysis (patients vs. procedures). A summary of the GRADE assessments by outcome domain is provided in Table 5.

**Table 5:** Certainty of evidence (GRADE) assessment across outcome domains.

Outcome domain	No. of studies	Study designs	Key findings (summary)	Certainty of evidence (GRADE)	Main reasons for downgrading
Overall complication rate (primary outcome)	1*	Prospective cohort	Low reported overall complication rate in the single study providing fully extractable numerical safety data; serious events <0.1%; no clear technique superiority	Low	Single quantitative dataset; observational study design; imprecision due to limited quantitative evidence; inability to assess consistency or heterogeneity across studies
Seroma	1*	Prospective cohort	Seroma was reported infrequently in the available quantitative dataset; however, qualitative evidence suggested a relatively higher frequency of seroma in RFAL cohorts	Low	Single quantitative dataset; small event numbers; observational design
Infection	1*	Prospective cohort	Infection was reported infrequently in the available quantitative dataset	Low	Single quantitative dataset; observational design; imprecision
Efficacy (fat removal/contour improvement)	9	RCTs + cohorts	Comparable fat removal and contour outcomes across modalities	Low	Indirect outcome measures; inconsistent reporting; lack of pooled analysis
Recovery outcomes (pain, bruising, downtime)	5	RCTs	WAL and PAL associated with faster early recovery	Low	Short-term follow-up; subjective outcomes
Skin tightening/contraction	6	RCTs + cohorts	Modest short-term tightening with LAL and RFAL; durability uncertain	Low	Inconsistent measurement; short follow-up
Long-term durability (>1 year)	4	Observational studies	Contour durability more dependent on weight stability than device	Very low	Limited long-term data; observational design; attrition

* The number of studies refers to those providing fully extractable numerical event counts and denominators for each safety endpoint. Although multiple studies reported safety outcomes, only one study provided sufficient numerical data for overall complications, seroma, and infection. Consequently, these outcomes are presented as descriptive quantitative findings, and pooled meta-analysis, heterogeneity assessment, and formal between-study comparisons were not performed.

### 3.6 Risk of bias assessment

**Figure 5:**
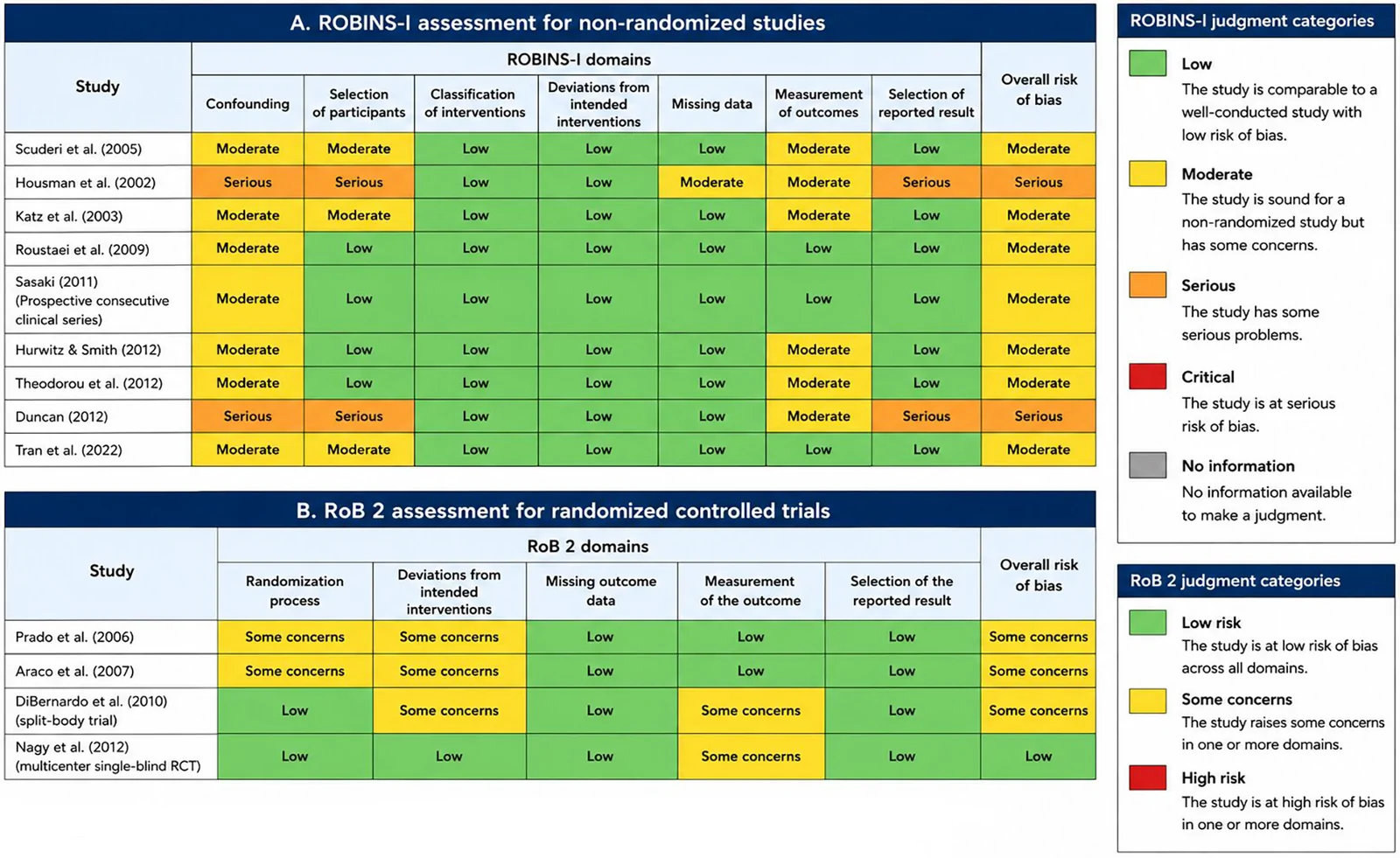
Risk of bias assessment **(A)** ROBINS-I assessment for non-randomized studies. **(B)** RoB 2 assessment for randomized controlled trials.

RoB 2 was used to evaluate risk of bias in randomized trials, and ROBINS-I was used to assess non-randomized studies. Following verification of the original study designs, Sasaki (19) was reclassified as a prospective consecutive clinical series and assessed using ROBINS-I. The assessments were repeated using the standard domain-level judgment rules for each tool. RoB 2 judgments were reported as low risk, some concerns, or high risk, whereas ROBINS-I judgments were reported as low, moderate, serious, critical, or no information. Because the tools use different domains and judgment frameworks, their results were not combined using a common numerical scale. Study-level domain judgments, overall assessments, and supporting reasons are provided in Supplementary Table S3. Graphical summaries of the separate RoB 2 and ROBINS-I assessments are presented in Figure 5.

## 4 Discussion

This systematic review and meta-analysis synthesized contemporary evidence comparing major liposuction techniques used exclusively in isolated cosmetic indications, addressing a long-standing gap in the literature where outcomes are often confounded by reconstructive cases or combined procedures. In contrast to previous systematic reviews that primarily emphasized complication rates or individual device categories, the present review integrated descriptive safety findings with a structured qualitative synthesis of efficacy, recovery, patient satisfaction, skin tightening, and longer-term durability to provide a broader assessment of clinically relevant outcomes across contemporary liposuction techniques. In 13 eligible studies over 20 years, all evaluated techniques, including SAL, PAL, UAL, LAL, WAL, and RFAL, were generally highly effective in fat removal and in contour improvement, and none of the methods reported a consistently superior result in total aspirate volume based on the available comparative evidence or available longer-term contour effect.

Comparative and randomized trials consistently indicated that the difference between methods has a greater impact on the operative efficiency and tissue handling, not on the quantity of fat removed. Studies generally reported that PAL was associated with improved aspiration efficiency and shorter operative duration. As previously noted, mechanical vibration improves surgeon ergonomics without affecting the final cosmetic appearance (2, 16). Studies generally reported that UAL and LAL facilitated fat emulsification and extraction, particularly in fibrous or secondary treatment areas. Beyond improved tissue handling, contemporary third-generation VASER systems may reduce intraoperative blood loss by facilitating selective adipose emulsification while minimizing injury to surrounding vascular structures. This potential advantage may be particularly relevant during large-volume liposuction procedures, although additional comparative studies remain warranted (4, 5, 19).

An additional source of heterogeneity was the variation in device platforms and technical protocols used across studies. Ultrasound-assisted liposuction investigations primarily evaluated VASER systems; however, details regarding probe configuration, ultrasound power settings, pulse mode, and treatment duration were frequently incompletely reported. Likewise, laser-assisted liposuction studies employed different laser platforms and wavelengths, while radiofrequency-assisted and water-assisted liposuction studies used manufacturer-specific systems with varying operative protocols. Consequently, differences in clinical outcomes may partly reflect variability in device settings and surgical technique rather than intrinsic differences between technologies.

Energy-based techniques demonstrated selective but limited advantages. Several studies reported modest short-term skin-tightening effects following LAL and RFAL, which have been attributed to thermal collagen contraction (5, 16, 20–22). However, consistent with limited available longitudinal observations, these benefits appeared to attenuate over time, with no consistent evidence of sustained difference in skin quality or contour durability at longer-term follow-up (20, 21). Among the available studies, RFAL cohorts generally reported the most consistent short-term skin-tightening effects across anatomical sites, particularly the abdomen, but at the cost of higher seroma incidence (21, 22). Interpretation of skin-tightening outcomes should consider the heterogeneity in assessment methods across studies, which included clinical photography, circumferential measurements, physician assessment, patient-reported outcomes, and, in a few studies, objective measurements of skin tightening. Additionally, newer skin-tightening technologies, such as argon plasma (Renuvion/J-Plasma) and Quantum RF, were not represented in the included studies. Therefore, the present findings are limited to the evaluated modalities (SAL, PAL, UAL, LAL, WAL, and RFAL) and should not be extrapolated to these newer platforms.

Recovery profiles varied more distinctly between techniques. Studies generally reported that WAL was associated with the lower incidence of early postoperative pain, ecchymosis, and shorter functional recovery, which contributes to its implementation in patients who value short-term comfort (7, 18). Although these differences were noted early, patient satisfaction remained consistently high (≥80% across modalities). However, satisfaction was assessed using heterogeneous patient-reported outcome instruments across the included studies, including structured satisfaction questionnaires, Likert-type rating scales, postoperative surveys, and patient interviews where reported. Because these assessment methods were not standardized, direct quantitative comparison between techniques was not feasible, and satisfaction outcomes were synthesized qualitatively. These findings are consistent with large observational studies demonstrating that patient satisfaction is influenced more by contour smoothness and appropriate expectation management than by the specific liposuction device used (15, 22).

The available quantitative safety evidence suggests that cosmetic liposuction is associated with a generally low rate of adverse events when performed in appropriately selected patients. However, because only one study provided fully extractable numerical safety data, while the remaining studies reported safety outcomes using heterogeneous study designs, units of analysis, and reporting methods, the reported complication frequencies should be interpreted as descriptive findings rather than definitive comparative estimates. The single study providing fully extractable numerical safety data reported low frequencies of overall complications, seroma, and infection. Serious complications were reported only rarely across the included studies. However, because study-level event counts and denominators were not consistently available, reliable estimation of their frequency or comparison between techniques was not possible. These observations are consistent with large cohort studies and national survey data reporting low rates of adverse events in SAL, UAL, and RFAL procedures (5, 15, 18). Recent evidence further reinforces the favorable safety profile of third-generation ultrasound-assisted liposuction (VASER). Tran et al. reported excellent safety and efficacy in a consecutive series of 261 patients undergoing VASER-assisted liposuction, with a very low incidence of postoperative complications (5). Furthermore, a large retrospective study involving 1,486 VASER-assisted liposuction procedures demonstrated that major complications remain uncommon when surgery is performed using standardized operative protocols, meticulous patient selection, and experienced surgical teams (24). More recent long-term evidence further demonstrated that contemporary ultrasound-assisted liposuction was associated with improved patient satisfaction, lower revision rates, more durable contour outcomes, and fewer clinically meaningful complications, highlighting the importance of careful patient selection and individualized aesthetic planning for sustained long-term success (25). Because quantitative safety evidence was available from only one study, confidence in these findings remains limited despite consistency with broader qualitative evidence. By comparison, the certainty of evidence for efficacy, recovery, skin tightening, and long-term durability was low to very low because of heterogeneity in study designs, variability in outcome definitions, limited follow-up, and reliance on observational evidence. Such limitations indicate that, despite the favorable safety profile of modern liposuction techniques, conclusions regarding comparative efficacy, recovery benefits, and long-term durability should be interpreted cautiously owing to the limited availability of high-quality comparative evidence. Qualitative evidence suggested that some studies reported lower complication rates following LAL, whereas observational studies reported a slightly higher frequency of minor complications with PAL and seroma with RFAL. However, these findings were based on a limited number of heterogeneous studies and should be interpreted as observational trends rather than definitive evidence of technique-specific superiority or inferiority. Direct comparative studies remain limited, precluding firm conclusions regarding the relative safety of individual modalities (16, 18, 21, 22).

Although quantitative synthesis was initially planned for selected safety endpoints, verification of the extracted data demonstrated that only one study provided fully extractable numerical event data. Accordingly, these safety outcomes are presented descriptively rather than as pooled estimates. This approach avoided combining non-equivalent units of analysis and ensured that each study was interpreted according to its original reporting framework. In addition, the limited number of studies providing extractable quantitative safety data and the inconsistency in technique-specific outcome reporting did not permit additional exploratory analyses beyond the predefined descriptive synthesis. Consequently, descriptive findings should be interpreted with appropriate caution. To provide an integrated overview of these heterogeneous findings, a structured qualitative evidence summary is presented in Table 6. Overall, the included studies reported comparable fat removal and contour improvement across all evaluated techniques when used in appropriate clinical settings. The available qualitative evidence suggested that energy-assisted modalities, particularly LAL and RFAL, may provide modest short-term skin-tightening benefits (17, 21, 22), whereas WAL was generally associated with more favorable early recovery, including reduced postoperative pain and ecchymosis (7, 19). Differences in complication profiles were small, with observational studies reporting a slightly higher incidence of seroma following RFAL and a modest increase in minor complications among PAL cohorts (16, 21, 22). Given the low to very low certainty of evidence for these outcomes, these findings should be interpreted as descriptive trends rather than definitive comparative advantages of individual liposuction techniques.

**Table 6:** Qualitative evidence summary of comparative clinical outcomes across contemporary cosmetic liposuction techniques.

Outcome Domain	SAL	PAL	UAL (VASER)	LAL	WAL	RFAL	Strength of evidence (GRADE)
Fat removal/contour improvement	Similar outcomes reported in the available comparative studies	Evidence from a single study suggested similar outcomes	Similar outcomes reported in the available comparative studies	Similar outcomes reported in the available comparative studies	No eligible evidence	Evidence from the available studies suggested similar outcomes	Low
Operative efficiency	Insufficient comparative evidence	Evidence suggests improved aspiration efficiency and shorter operative time	Evidence suggests improved tissue handling, particularly in fibrous areas	Evidence suggests facilitated fat emulsification	No eligible evidence	No eligible evidence	Low
Early recovery	Evidence from the available studies was limited	Evidence from a single study was limited	No eligible evidence	No eligible evidence	Evidence suggests less postoperative pain, bruising, and faster early recovery	No eligible evidence	Low
Short-term skin tightening	Limited evidence; no consistent benefit demonstrated	No eligible evidence	Limited evidence; no consistent benefit demonstrated	Evidence suggests modest short-term improvement	No eligible evidence	Evidence suggests greater short-term improvement in the available studies	Low
Overall safety	Generally favorable safety profile reported	Observational evidence suggested a slightly higher frequency of minor complications	Generally favorable safety profile reported	Generally favorable safety profile reported	No eligible evidence	Observational evidence suggested a slightly higher frequency of seroma	Low
Longer-term contour durability (1–4 years)	Limited evidence; no consistent differences reported	Limited evidence; no consistent differences reported	Limited evidence; no consistent differences reported	Limited evidence; no consistent differences reported	No eligible evidence	No eligible evidence	Very Low

The available evidence suggested that longer-term outcomes were broadly comparable across techniques in the limited studies reporting extended follow-up. However, claims regarding durability beyond short- to mid-term follow-up (approximately 1–4 years) were supported by a small number of observational studies and should therefore be interpreted cautiously. Where longer-term stability was reported, the available evidence suggested that postoperative weight control exerted a greater influence on contour durability than device selection (15). Although liposuction is performed primarily for aesthetic body contouring rather than weight reduction, recent studies of VASER-assisted liposuction have reported modest postoperative reductions in body weight and body mass index, particularly when combined with sustained lifestyle modification. Beyond cosmetic outcomes, emerging evidence has also investigated the metabolic consequences of ultrasound-assisted liposuction, including changes in lipid profiles, glucose homeostasis, and insulin sensitivity. While some studies have demonstrated transient improvements in serum lipid parameters and inflammatory markers following adipose tissue removal (26), randomized and longitudinal investigations indicate that liposuction alone does not consistently improve insulin resistance or glucose metabolism (27). These findings indicate that any observed metabolic benefits associated with VASER-assisted liposuction may be secondary to postoperative weight maintenance and healthy behavioral changes rather than the surgical procedure itself. This qualitative synthesis complements the descriptive safety findings and provides a contextual, rather than definitive, framework for interpreting technique-specific strengths where formal quantitative pooling was not supported by the available evidence.

## 5 Limitations of the study

There are a number of limitations that should be noted. To begin with, heterogeneous study designs, definition of outcomes, and duration of follow-up restricted the extent of quantitative synthesis on outcomes other than safety. Second, a number of the studies were observational, which creates the possibility of selection bias and confounding despite risk-of-bias evaluation. Furthermore, only one study provided fully extractable numerical safety data, preventing formal quantitative synthesis and limiting the strength of conclusions regarding complication rates. Although inclusion criteria were predefined, this variability may have influenced the precision of the reported safety findings and limits direct comparative interpretation across techniques. Third, study-level reporting of long-term outcomes was inconsistent, with relatively few studies providing follow-up beyond 12–36 months and only very limited evidence extending beyond approximately 5 years, particularly for newer energy-based technologies. Lastly, aesthetic outcomes and satisfaction indicators were frequently subjective and variably reported, precluding standardized pooled analysis and formal certainty grading across several outcome domains.

## 6 Future perspectives

Well-powered randomized controlled trials directly comparing liposuction techniques using standardized outcome measures, including objective three-dimensional imaging and validated patient-reported outcome instruments, are needed. Long-term (>5–10 years) follow-up studies are required to better characterize contour durability and skin quality, particularly for energy-assisted modalities. In addition, emerging hybrid technologies combining mechanical and energy-based approaches warrant evaluation in strictly defined cosmetic populations.

## 7 Conclusion

This systematic review provides a comprehensive synthesis of contemporary evidence from isolated cosmetic liposuction procedures and indicates that modern liposuction techniques are generally associated with favorable safety profiles in cosmetic body contouring, based primarily on descriptive safety reporting together with structured qualitative synthesis. Evidence regarding efficacy, patient satisfaction, recovery, skin tightening, and long-term durability was derived primarily from qualitative synthesis because of substantial heterogeneity in outcome definitions and reporting across studies. The available evidence suggests that any observed differences among modalities primarily relate to operative efficiency, early recovery, and short-term skin-tightening effects, whereas evidence regarding longer-term contour durability and skin quality remains limited and insufficient to support definitive comparative conclusions. However, these observations should be interpreted cautiously because of study heterogeneity, limited direct comparative evidence, and the predominance of observational data. Given unresolved heterogeneity and limited stratified evidence, technique selection should remain individualized, guided by patient anatomy, recovery priorities, and surgeon expertise rather than expectations of superior fat removal, long-term satisfaction, or definitive superiority of any individual modality.
