# Supplementary material for: Safety and clinical outcomes of contemporary liposuction techniques: evidence from a systematic review
Source: Front Surg. 2026 Sep 7;13:1892614. doi: 10.3389/fsurg.2026.1892614 (PMC13591073; doi:10.3389/fsurg.2026.1892614)
Supplement: Supplementary file 1 [file Datasheet1.docx]

**Table S1. Electronic Search Strategies**

| Database | Time Frame | Search Strategy |
| --- | --- | --- |
| PubMed (MEDLINE) | January 2000 – 15 March 2025 | (“cosmetic liposuction” OR “aesthetic liposuction” OR lipoplasty OR lipectomy) AND (“suction-assisted” OR “power-assisted” OR “ultrasound-assisted” OR “laser-assisted” OR “water-assisted” OR “radiofrequency-assisted” OR SAL OR PAL OR UAL OR LAL OR WAL OR RFAL) AND (complication* OR safety OR outcome* OR satisfaction OR efficacy) |
| EMBASE | January 2000 – 15 March 2025 | (‘cosmetic liposuction’ OR ‘aesthetic liposuction’ OR lipoplasty OR lipectomy) AND (‘suction assisted’ OR ‘power assisted’ OR ‘ultrasound assisted’ OR ‘laser assisted’ OR ‘water assisted’ OR ‘radiofrequency assisted’ OR SAL OR PAL OR UAL OR LAL OR WAL OR RFAL) AND (complication* OR safety OR outcome* OR satisfaction OR efficacy) |
| Web of Science (Core Collection) | January 2000 – 15 March 2025 | (“cosmetic liposuction” OR “aesthetic liposuction” OR lipoplasty OR lipectomy) AND (“suction assisted” OR “power assisted” OR “ultrasound assisted” OR “laser assisted” OR “water assisted” OR “radiofrequency assisted” OR SAL OR PAL OR UAL OR LAL OR WAL OR RFAL) AND (complication* OR safety OR outcome* OR satisfaction OR efficacy) |

**Table S2. Availability of study-level safety data for quantitative and qualitative synthesis, with the original unit of analysis retained for each included study**

| **Outcome** | **Included study** | **Study design** | **Original unit of analysis** | **Denominator reported** | **Reported events** | **Contribution to synthesis** |
| --- | --- | --- | --- | --- | --- | --- |
| Overall complications | Housman et al., 2002 | National survey | Procedures | 66,570 procedures | Overall complication rates, serious adverse events, and mortality reported; individual study-level event counts not fully extractable | Qualitative synthesis |
| Overall complications | Katz et al., 2003 | Retrospective comparative cohort | Patients | 207 patients | Systemic and local complications reported; study-level event counts not extractable for pooled analysis | Qualitative synthesis |
| Overall complications | Araco et al., 2007 | Randomized controlled trial | Patients | 60 patients | Early postoperative safety outcomes reported; no overall complication count available | Qualitative synthesis |
| Overall complications | Roustaei et al., 2009 | Prospective cohort | Procedures | 660 procedures | Numerical complication data reported | Quantitative synthesis |
| Overall complications | Sasaki, 2011 | Prospective consecutive clinical series | Patients | 41 patients | Safety outcomes reported qualitatively without extractable pooled event counts | Qualitative synthesis |
| Overall complications | Nagy et al., 2012 | Multicenter randomized controlled trial | Patients | 20 patients | Adverse events reported; numerical complication counts not fully extractable | Qualitative synthesis |
| Overall complications | Hurwitz & Smith, 2012 | Prospective cohort | Patients | 17 patients | Safety outcomes reported; numerical event counts insufficient for pooling | Qualitative synthesis |
| Overall complications | Theodorou et al., 2012 | Prospective cohort | Patients | 97 patients | Minor complications and safety outcomes reported; incomplete numerical event reporting | Qualitative synthesis |
| Overall complications | Duncan, 2012 | Prospective pilot study | Patients | 12 patients | Safety outcomes reported; complication counts not extractable | Qualitative synthesis |
| Overall complications | Tran et al., 2022 | Retrospective cohort | Patients | 261 patients | Overall safety reported; individual complication counts incompletely extractable | Qualitative synthesis |
| Seroma | Roustaei et al., 2009 | Prospective cohort | Procedures | 660 procedures | Numerical seroma events reported | Quantitative synthesis |
| Seroma | Theodorou et al., 2012 | Prospective cohort | Patients | 97 patients | Seroma reported descriptively; numerical counts not fully extractable | Qualitative synthesis |
| Infection | Roustaei et al., 2009 | Prospective cohort | Procedures | 660 procedures | Numerical infection events reported | Quantitative synthesis |
| Infection | Tran et al., 2022 | Retrospective cohort | Patients | 261 patients | Infection assessed as part of overall safety; separate numerical counts not extractable | Qualitative synthesis |

**Abbreviations:** RCT, randomized controlled trial.

**The unit of analysis shown in this table corresponds to that reported in the original publication. Patient-level denominators were preferentially interpreted where available; however, studies reporting outcomes per procedure (e.g., national surveys and prospective procedural cohorts) retained procedure-level denominators. Where reported, split-body studies were interpreted separately from patient-level outcomes and were not combined quantitatively. Only Roustaei et al. (2009) provided fully extractable numerical safety data for these endpoints. Consequently, pooled meta-analysis and heterogeneity statistics were not performed, and the findings are presented descriptively.**

**Table S3. Study-level Risk-of-Bias Assessment**

| **Study** | **Study Design** | **Assessment Tool** | **Randomization Process / Confounding** | **Deviations from Intended Intervention** | **Missing Outcome Data** | **Outcome Measurement** | **Selection of Reported Results** | **Overall Risk of Bias** |
| --- | --- | --- | --- | --- | --- | --- | --- | --- |
| Prado et al. (2006) | Randomized controlled trial | RoB 2 | Low | Some concerns (blinding not fully described) | Low | Low | Low | Some concerns |
| Araco et al. (2007) | Randomized controlled trial | RoB 2 | Some concerns (randomization details incompletely reported) | Some concerns | Low | Low | Low | Some concerns |
| DiBernardo et al. (2010) | Randomized split-body trial | RoB 2 | Low | Some concerns (split-body design; blinding limited) | Low | Some concerns (subjective aesthetic outcomes) | Low | Some concerns |
| Nagy et al. (2012) | Multicenter randomized single-blind trial | RoB 2 | Low | Low | Low | Some concerns | Low | Low |
| Scuderi et al. (2005) | Comparative clinical study | ROBINS-I | Moderate | Low | Low | Moderate | Low | Moderate |
| Housman et al. (2002) | National survey | ROBINS-I | Serious (selection and reporting bias) | Low | Moderate | Moderate | Moderate | Serious |
| Katz et al. (2003) | Retrospective comparative cohort | ROBINS-I | Moderate | Low | Low | Moderate | Low | Moderate |
| Roustaei et al. (2009) | Prospective cohort | ROBINS-I | Moderate | Low | Low | Low | Low | Moderate |
| Sasaki (2011) | Prospective consecutive clinical series | ROBINS-I | Moderate | Low | Low | Low | Low | Moderate |
| Hurwitz & Smith (2012) | Prospective cohort | ROBINS-I | Moderate | Low | Low | Moderate | Low | Moderate |
| Theodorou et al. (2012) | Prospective cohort | ROBINS-I | Moderate | Low | Low | Moderate | Low | Moderate |
| Duncan (2012) | Prospective pilot study | ROBINS-I | Serious (small sample, no comparator) | Low | Low | Moderate | Moderate | Serious |
| Tran et al. (2022) | Retrospective cohort | ROBINS-I | Moderate | Low | Low | Low | Low | Moderate |
